# Gut Microbiome–Sleep Crosstalk: Mechanistic Pathways, Dysbiosis Signatures, and Microbiome‐Based Interventions

**DOI:** 10.1002/brb3.71525

**Published:** 2026-06-04

**Authors:** Ghaleb Oriquat, Shaker Al‐Hasnaawei, Hayjaa Mohaisen Mousa, Malathi H, Siya Singla, Samir Sahoo, Vimal Arora, Ashish Singh Chauhan, Mehrdad Nourizadeh

**Affiliations:** ^1^ Faculty of Allied Medical Sciences, Hourani Center for Applied Scientific Research Al‐Ahliyya Amman University Amman Jordan; ^2^ College of Pharmacy Islamic University Najaf Iraq; ^3^ Department of Medical Analysis, Medical Laboratory Technique College The Islamic University of Al Diwaniyah Al Diwaniyah Iraq; ^4^ Department of Medicinal Chemistry Al‐Turath University Baghdad Iraq; ^5^ Department of Biotechnology and Genetics, School of Sciences JAIN (Deemed to Be University) Bangalore Karnataka India; ^6^ Centre for Research Impact and Outcome, Chitkara University Institute of Engineering and Technology Chitkara University Rajpura Punjab India; ^7^ Department of General Medicine IMS and SUM Hospital Siksha ‘O’ Anusandhan (Deemed to Be University) Bhubaneswar Odisha India; ^8^ University Institute of Pharma Sciences Chandigarh University Mohali Punjab India; ^9^ Uttaranchal Institute of Pharmaceutical Sciences, Division of Research and Innovation Uttaranchal University Dehradun Uttarakhand India; ^10^ Neurosciences Research Center Tabriz University of Medical Sciences Tabriz Iran

**Keywords:** dysbiosis, gut–brain axis, neuroinflammation, psychobiotics, short‐chain fatty acids, sleep disturbances

## Abstract

**Background:**

This review examines the bidirectional relationship between sleep regulation and the gut microbiome within the gut–brain axis, with particular attention to mechanistic pathways, disorder‐associated dysbiosis patterns, and microbiome‐targeted interventions in insomnia, obstructive sleep apnea, circadian disruption, and sleep loss‐related states.

**Methods:**

We critically synthesized evidence from both human and preclinical studies, focusing on microbial metabolites, neuroimmune and neuroendocrine signaling, circadian regulation, and intervention‐based approaches. Rather than only summarizing individual studies, we aimed to distinguish associative human findings from mechanistic evidence derived mainly from animal models.

**Results:**

Current evidence supports a bidirectional link between sleep and the gut microbiome. Microbiota‐derived metabolites, particularly short‐chain fatty acids, tryptophan‐related metabolites, and gamma‐aminobutyric acid, appear to influence sleep homeostasis through effects on intestinal barrier integrity, inflammatory tone, stress‐axis regulation, and central signaling pathways. Across sleep disorders, recurrent microbial patterns include reduced abundance of potentially beneficial taxa such as *Bifidobacterium* and *Faecalibacterium* and enrichment of pro‐inflammatory or stress‐associated taxa, although these signatures are not yet fully consistent across cohorts or disorders. In humans, most data remain observational and support association rather than causation, whereas stronger mechanistic support comes from experimental models of sleep deprivation, intermittent hypoxia, and microbiota transfer. Early intervention studies suggest that selected probiotics, prebiotics, dietary modulation, and related microbiome‐directed strategies may improve sleep‐related outcomes, but the magnitude and reproducibility of these effects remain uncertain.

**Conclusion:**

The gut microbiome represents a promising mechanistic and therapeutic target in sleep medicine, but clinical translation is still constrained by heterogeneity in microbiome profiling, sleep phenotyping, intervention design, and strain‐specific effects. Future work should prioritize longitudinal human studies, standardized outcome measures, and mechanistically informed trials capable of identifying clinically actionable and biologically credible microbiome signatures.

## Graphical abstract

Gut dysbiosis and sleep disturbances interact bidirectionally through microbial metabolites, immune activation, and neuroendocrine signaling along the gut–brain axis. Microbiome‐based interventions, including probiotics, prebiotics, synbiotics, postbiotics, and fecal microbiota transplantation, may help improve sleep quality and reduce inflammatory burden.

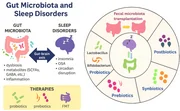

**Abbreviations**
BBB: blood–brain barrier
CBT‐I: cognitive behavioral therapy for insomnia
CNS: central nervous system
CPAP: continuous positive airway pressure
FMT: fecal microbiota transplantation
GABA: gamma‐aminobutyric acid
GOS: galactooligosaccharides
HPA axis: hypothalamic–pituitary–adrenal axis
IH: intermittent hypoxia
IL: interleukin
LPS: lipopolysaccharide
NREM: non‐rapid eye movement
OSA: obstructive sleep apnea
RCT: randomized controlled trial
SCFAs: short‐chain fatty acids
SD: sleep deprivation
TNF‐α: tumor necrosis factor alpha
TRF: time‐restricted feeding

## 1 Introduction

Sleep is a fundamental biological process that supports immune competence, emotional regulation, metabolic homeostasis, cognitive performance, and long‐term neurological health. Disturbances such as obstructive sleep apnea (OSA), chronic insomnia, circadian rhythm disruption, and recurrent sleep restriction affect a substantial proportion of the adult population and are increasingly recognized as contributors to cardiometabolic, psychiatric, and neurocognitive morbidity (Flikkema 2022). Although conventional models of sleep pathology have mainly focused on central neural circuitry, neurotransmitter imbalance, and endocrine dysregulation, recent work has expanded this framework by implicating the gut microbiome as a biologically relevant component of sleep regulation (Z. Wang et al. 2022).

The gut microbiome consists of a dynamic community of bacteria, viruses, fungi, and other microorganisms that interact continuously with the host through metabolic, immune, endocrine, and neural signaling. Through the gut–brain axis, microbial activity can influence central nervous system (CNS) function, while host sleep–wake rhythms, feeding behavior, stress reactivity, and circadian timing can in turn reshape the intestinal microbial ecosystem (Han et al. 2022). This bidirectional perspective has become increasingly important because it suggests that the microbiome may function not only as a correlate of disturbed sleep, but also as a potential contributor to sleep‐related pathophysiology in at least some contexts (Tian et al. 2022).

Interest in the microbiome‐sleep relationship has grown rapidly over the past few years. Human studies have linked poor sleep quality and sleep disorders with altered microbial diversity, depletion of potentially beneficial taxa, and enrichment of inflammatory or stress‐associated microbial communities (Lin et al. 2024; Mairinger et al. 2023). Experimental models have added mechanistic support by showing that sleep deprivation (SD), intermittent hypoxia (IH), and circadian misalignment can disrupt gut barrier integrity, alter microbial metabolites, and amplify systemic and neuroinflammatory signaling (Santos and Galiè 2024; Sen et al. 2021). At the same time, early interventional studies have begun to explore whether probiotics, prebiotics, dietary modulation, and related microbiome‐directed strategies can improve sleep outcomes. However, much of the current literature remains heterogeneous, and the field still faces major uncertainty regarding causality, reproducibility, and clinical translatability (Li et al. 2023; Singh and Negi 2025). A central challenge is that the available evidence is uneven in both design and interpretability. Most human studies remain observational and identify associations rather than causal relationships, whereas stronger mechanistic claims often rely on preclinical models that may not fully capture the complexity of human sleep disorders. In addition, findings are shaped by substantial variation in microbiome sampling methods, sequencing platforms, dietary background, medication exposure, sleep phenotyping, and participant comorbidities. These issues make it difficult to determine whether specific microbial signatures are truly disorder‐related, broadly stress‐responsive, or secondary to lifestyle and metabolic factors that commonly accompany sleep disruption (Bautista et al. 2025; Wu et al. 2026).

This review therefore aims to critically synthesize, rather than simply catalog, current evidence on gut microbiome–sleep crosstalk. We focus on core mechanistic pathways, disorder‐specific dysbiosis signatures, evidence from SD and experimental models, microbial chronobiology, and microbiome‐targeted interventions, while also addressing the translational barriers that currently limit clinical application. Particular attention is given to separating human associative evidence from mechanistic insights derived from animal studies, since this distinction is essential for a balanced interpretation of the field and for identifying realistic priorities for future research.

## 2 Core Mechanistic Pathways

The gut–brain axis is a bidirectional communication network linking the gastrointestinal tract and the CNS through neural, endocrine, immune, and metabolic signaling. In sleep physiology, this framework is increasingly viewed as more than a background regulatory system. It provides a biologically plausible route through which microbial metabolites, intestinal barrier integrity, inflammatory tone, and host neuroendocrine responses may influence sleep initiation, sleep continuity, and circadian alignment (Akpınar 2018). This shift in framing is important because it moves the field away from treating the microbiome as a passive correlate of health and toward evaluating its potential role in sleep‐related pathophysiology (Lin et al. 2024).

### 2.1 Tryptophan Metabolism and Serotonergic–Melatonergic Signaling

One of the most frequently discussed mechanisms involves microbial regulation of tryptophan metabolism. Although tryptophan is derived from the diet, its downstream fate is shaped by host–microbial interactions within the intestine. Rather than affecting only a single conversion step, the microbiota helps influence how tryptophan is distributed across serotonin‐related, kynurenine‐related, and indole‐producing pathways, each of which may have different consequences for sleep and circadian biology (Strandwitz 2018). Yano et al. (2015) showed that colonization with specific spore‐forming bacteria restored peripheral serotonin biosynthesis in germ‐free mice, supporting the idea that commensal microbes can shape host serotonergic signaling. Because serotonin contributes to sleep–wake regulation and also serves as a precursor for melatonin synthesis, microbiome‐dependent alterations in tryptophan handling may influence sleep through both circadian and neurochemical pathways (Hwang and Oh 2025). At the same time, most human evidence remains indirect, and it is still unclear to what extent peripheral microbial effects on tryptophan metabolism translate into clinically meaningful sleep changes (Lee et al. 2024; Lin et al. 2024; Sutanto et al. 2024).

### 2.2 Microbial Neuroactive Compounds and Gamma‐Aminobutyric Acid (GABA)‐Related Signaling

A second pathway involves microbial modulation of neuroactive compounds, particularly GABA. *Lactobacillus* and *Bifidobacterium* species are frequently cited for their capacity to produce GABA, which is the principal inhibitory neurotransmitter involved in reducing arousal and promoting non‐rapid eye movement (NREM) sleep (Das et al. 2025). Experimental studies suggest that GABA‐producing strains can shorten sleep latency and improve sleep quality in mice (Samtiya et al. 2022; Sejbuk et al. 2024). However, these findings should not be interpreted as evidence that microbially derived GABA simply enters the brain and directly replaces host neurotransmission. A more plausible interpretation is that microbial neuroactive signaling influences sleep indirectly through enteric pathways, vagal afferents, immune modulation, and downstream changes in host neuroendocrine function. This distinction matters because it separates mechanistic plausibility from overly simplified claims of direct neurotransmitter substitution (Faysal et al. 2025; Grant et al. 2026; Seong et al. 2024).

### 2.3 Gut Barrier Dysfunction, Immune Signaling, and Neuroinflammatory Transmission

Barrier dysfunction provides another major mechanistic route by which dysbiosis may affect sleep. When microbial composition becomes unstable, intestinal tight junction integrity can weaken, allowing greater translocation of lipopolysaccharide (LPS), peptidoglycan, and other microbial products into the circulation (Pan et al. 2023). These signals activate pattern‐recognition pathways, including toll‐like receptors such as TLR4, and promote production of pro‐inflammatory mediators including tumor necrosis factor alpha (TNF‐α), interleukin (IL)‐1β, and IL‐6, cytokines known to influence sleep continuity, slow‐wave activity, and arousal thresholds (Golshah et al. 2024; Tahara et al. 2018; Zielinski et al. 2017).

Peripheral inflammatory signaling may then reach the brain through several partially overlapping routes. Cytokines can act at the level of the blood–brain barrier (BBB), signal through endothelial and perivascular cells, or engage afferent neural pathways including the vagus nerve. These processes can prime or activate glial cells in sleep‐relevant regions such as the hypothalamus and brainstem. Microglial activity in the preoptic area has been associated with reduced NREM sleep and increased wake‐promoting tone, supporting the idea that gut‐derived immune activation may alter sleep architecture through central neuroimmune mechanisms rather than through a nonspecific systemic stress response alone (Mahalakshmi et al. 2022; Zielinski et al. 2017). A point that should be made clearly is that peripheral inflammation and central neuroinflammation are not interchangeable. The transition from intestinal barrier dysfunction to altered sleep requires biological relay systems, not just the presence of inflammation in general (Malita et al. 2025; Tang et al. 2026).

### 2.4 Short‐Chain Fatty Acids (SCFAs), Epigenetic Modulation, and Neuroimmune Restraint

SCFAs, especially butyrate, acetate, and propionate, represent another important mechanistic link between the gut microbiome and sleep. These metabolites are generated through bacterial fermentation of dietary fiber and exert broad effects on intestinal physiology, host metabolism, and immune regulation (Deleu et al. 2021). Among them, butyrate has drawn particular attention because of its role in maintaining epithelial integrity, limiting inflammatory signaling, and modulating neuroimmune pathways relevant to sleep. Experimental work suggests that butyrate can increase NREM sleep, reduce hypothalamic inflammatory mediators such as IL‐6 and TNF‐α, and alter microglial activation states (Fukushima 2022; Szentirmai et al. 2019; Wang et al. 2024).

Butyrate may also influence inflammatory gene expression through histone deacetylase inhibition, which links microbial metabolism to epigenetic regulation of immune and neural responses (Sun et al. 2019). What matters mechanistically is that butyrate is unlikely to act through a single pathway. Its sleep‐related effects may reflect simultaneous reinforcement of the intestinal barrier, reduction of systemic endotoxemia, modulation of cytokine tone, and epigenetic effects on immune and neural cells. This multilayer interpretation is much stronger than describing SCFAs as generic beneficial metabolites, because it explains how loss of SCFA‐producing taxa may affect both peripheral and central sleep‐related biology. It is also important to acknowledge that the evidence remains much stronger in animal models than in human sleep cohorts (Magzal et al. 2021; Yerlikaya et al. 2025).

### 2.5 Vagal and Enteroendocrine Communication

Neural signaling through the vagus nerve provides a more rapid route of gut–brain communication. Signals generated by microbial metabolites, enteroendocrine cells, and immune mediators can activate vagal afferents and thereby influence central circuits involved in arousal, stress responsivity, and autonomic balance. Work with selected probiotic strains suggests that vagal signaling may be required for at least some microbiome‐related behavioral and neurochemical effects (Bravo et al. 2011). In the context of sleep research, this supports a model in which microbial activity modifies host sleep indirectly through neural relays rather than through one circulating metabolite acting alone. This framework also helps explain why strain‐specific effects are common, since different organisms are unlikely to engage the same host sensory, immune, and neuroendocrine pathways to the same extent (Cheng et al. 2025; Teichman et al. 2020).

### 2.6 Hypothalamic–Pituitary–Adrenal (HPA) Axis Reactivity and Stress‐Linked Sleep Vulnerability

**FIGURE 1:**
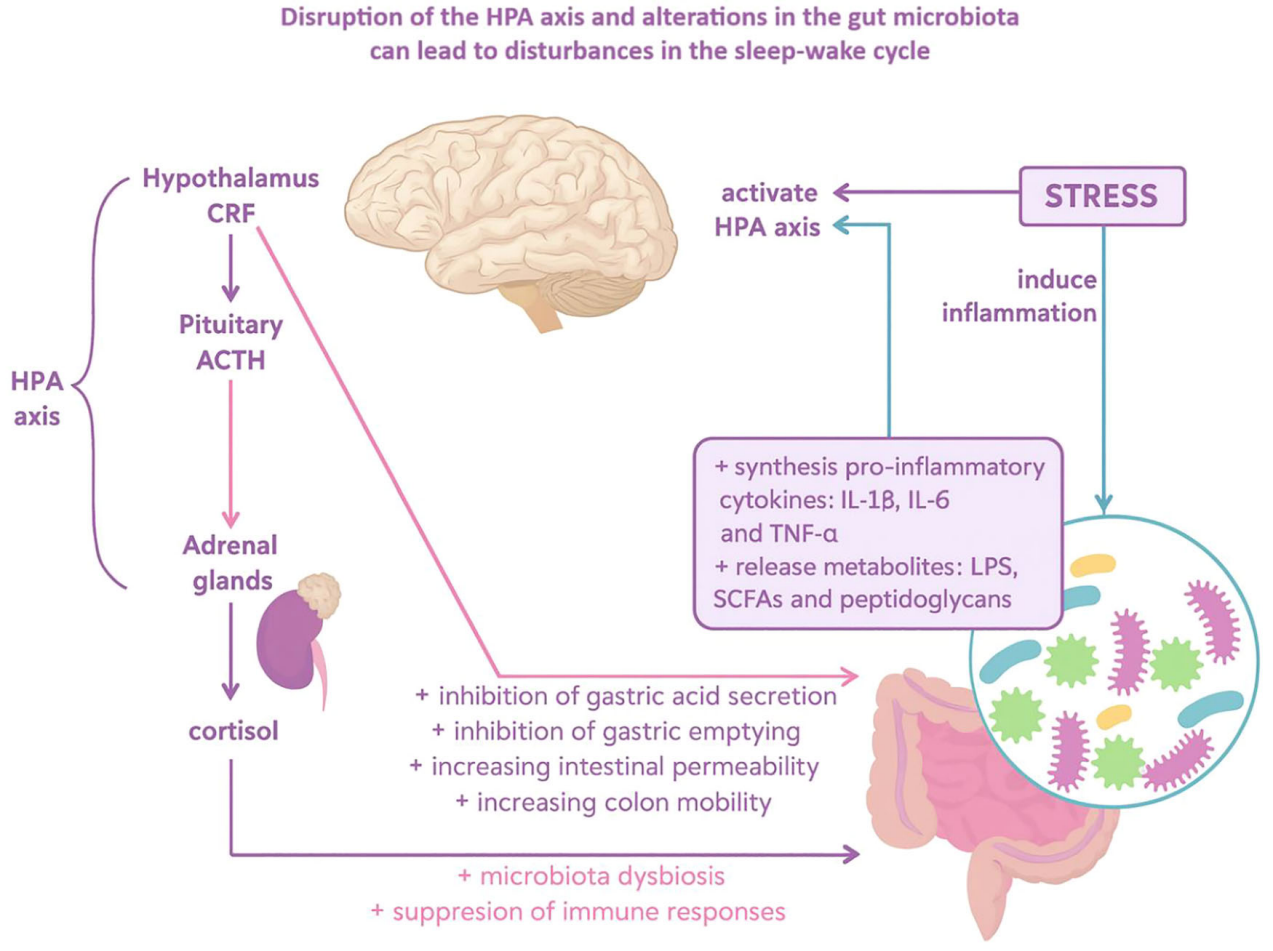
HPA axis‐mediated interaction between stress, gut dysbiosis, inflammation, and sleep–wake disturbance. Stress‐related activation of the hypothalamic–pituitary–adrenal axis may alter cortisol signaling, intestinal permeability, gastrointestinal motility, immune responses, and microbial composition. These changes can promote inflammatory mediator release and microbial metabolite imbalance, thereby contributing to disrupted sleep–wake regulation.

The gut microbiome may also influence sleep through its effects on HPA axis regulation. Dysbiosis, especially when accompanied by increased intestinal permeability and low‐grade inflammation, may contribute to heightened cortisol signaling, prolonged sleep latency, and reduced slow‐wave sleep. This pathway is particularly relevant in stress‐sensitive sleep phenotypes, where neuroendocrine reactivity may be as important as sleep duration itself (Maki et al. 2026) (Figure 1).

Selected probiotic strains, including *Bifidobacterium longum* and *Lactobacillus helveticus*, have been reported to reduce HPA axis reactivity and improve stress‐related behavioral or sleep‐associated outcomes in both animal and human studies (Messaoudi et al. 2011; Rosin et al. 2021). However, reduced stress reactivity should not automatically be interpreted as direct improvement in objective sleep physiology. This is an important distinction because some interventions may improve stress resilience more clearly than they improve polysomnographic sleep measures. A more precise interpretation is that the microbiome may shape sleep vulnerability under stress‐related conditions by modulating barrier integrity, inflammatory tone, and neuroendocrine responsiveness rather than acting as a universal regulator across all sleep disorders (Beck et al. 2022; Chichlowski et al. 2022; Lin et al. 2024).

### 2.7 An Integrated Bidirectional Model

**FIGURE 2:**
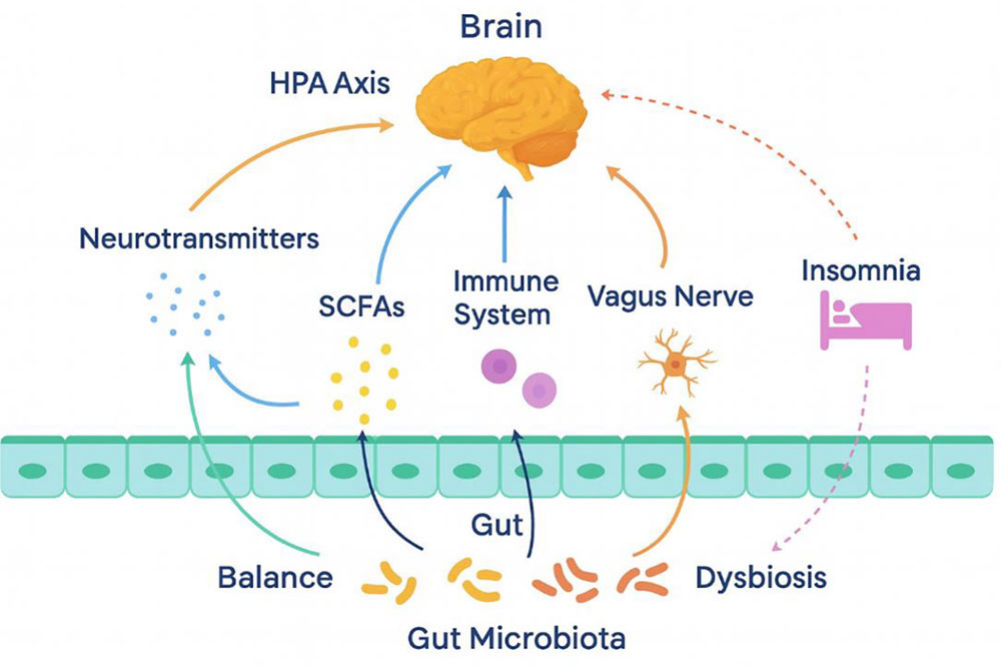
Integrated microbiota–gut–brain pathways linking microbial balance and dysbiosis to sleep regulation and insomnia. Gut microbiota may influence sleep‐related brain function through microbial metabolites such as short‐chain fatty acids, neuroactive compounds, immune signaling, vagal communication, and hypothalamic–pituitary–adrenal axis regulation. Conversely, sleep disturbance and insomnia may worsen gut dysbiosis, creating a bidirectional feedback loop between microbial imbalance and altered sleep physiology.

These mechanisms should not be viewed in isolation. Current evidence instead supports an integrated model in which microbial metabolites, epithelial barrier integrity, immune activation, neuroactive signaling, vagal communication, and stress‐axis regulation interact dynamically. Sleep disturbance can disrupt microbial composition and function, while microbial dysfunction can in turn amplify inflammation, metabolic instability, stress responsivity, and altered sleep signaling. This bidirectional architecture is one reason why causality remains difficult to establish in human studies, even when the biological rationale is strong. What can be stated more confidently is that the microbiome–sleep relationship is mechanistically layered, biologically plausible, and supported most strongly by preclinical evidence, whereas the strength and direction of these effects in humans still require more precise definition (Figure 2).

## 3 Disorder‐Specific Dysbiosis Signatures

Emerging evidence suggests that sleep disorders are accompanied by measurable alterations in gut microbial composition and function. Across clinical studies, these alterations often include reduced microbial diversity, depletion of taxa linked to barrier support and SCFA production, and enrichment of taxa associated with inflammatory signaling or metabolic stress. However, these patterns should not yet be interpreted as stable or disease‐defining enterotypes, because most available studies are cross‐sectional, relatively small, and influenced by major confounders such as diet, obesity, medication exposure, psychiatric symptoms, and host circadian behavior. For this reason, the most defensible interpretation at present is that sleep disorders are associated with recurring but not fully standardized dysbiosis patterns (Pala et al. 2024; Romanenko et al. 2025; Zeng et al. 2025).

### 3.1 Insomnia

Insomnia has been linked to reproducible, although not entirely uniform, microbiome alterations. Several studies report reduced abundance of potentially beneficial genera such as *Faecalibacterium*, *Lactobacillus*, and *Bifidobacterium*, together with relative expansion of Proteobacteria and other taxa commonly associated with inflammatory or stress‐related states. Because some of the depleted organisms contribute to SCFA generation and mucosal homeostasis, these observations are biologically consistent with a model in which chronic sleep disturbance is accompanied by impaired barrier support and heightened inflammatory tone (Park et al. 2026; Xu et al. 2026).

At the same time, the human insomnia literature remains primarily associative rather than causal. Reported taxa differ across cohorts, and some microbial shifts may reflect stress exposure, altered diet, reduced physical activity, or psychiatric comorbidity rather than insomnia itself. Studies stratifying patients by HPA axis reactivity have identified taxa such as *Bacteroides* and *Collinsella*, raising the possibility that microbial changes in insomnia may be more tightly linked to stress physiology and neuroendocrine context than to sleep loss alone (Pacheco et al. 2024; Yan et al. 2025). This is an important distinction because it argues against presenting a single insomnia‐specific microbial signature as already established.

### 3.2 Circadian Rhythm Disruption

Circadian disruption, including shift work and jet lag‐related misalignment, is associated with microbial instability and altered temporal organization of the gut microbiome. Available human and preclinical studies suggest that this phenotype may be accompanied by enrichment of taxa such as Enterobacteriaceae and by broader shifts in inflammatory or stress‐related microbial functions (Thaiss et al. 2014). Experimental light–dark reversal models have also shown that circadian disturbance can coincide with altered microbial composition and impaired host–microbe synchrony (Leone et al. 2015; Tian et al. 2022).

At this stage, the most defensible conclusion is that circadian disruption is associated with a dysregulated microbial state rather than with a single reproducible taxonomic signature. This point is particularly important because time‐of‐sampling, feeding schedule, and host behavioral context can strongly influence microbiome readouts in circadian studies (Teichman et al. 2020; Withrow et al. 2021).

### 3.3 OSA

OSA is increasingly associated with gut dysbiosis characterized by enrichment of genera such as *Prevotella*, *Streptococcus*, and *Actinomyces*, together with relative depletion of taxa often linked to anti‐inflammatory and barrier‐supporting functions, including *Faecalibacterium*. In clinical cohorts, these microbial patterns have been associated with inflammatory burden, cardiometabolic stress, and neurocognitive symptoms, although the degree of consistency varies across studies (Baldanzi et al. 2022; Ko et al. 2019; Ko et al. 2019). Obesity, insulin resistance, and dietary habits are particularly important confounders in OSA cohorts and may partly shape the observed microbial profile (Gentile et al. 2025; Mou et al. 2024).

Compared with insomnia, OSA has somewhat stronger mechanistic support from experimental models, but the human signature itself is still best interpreted as an association rather than a definitive biomarker panel. IH and sleep fragmentation likely contribute to the observed dysbiosis, yet it remains difficult to determine how much of the microbial pattern is driven by apnea‐related physiology itself and how much reflects the broader metabolic context in which OSA commonly occurs (Karthik S et al. 2026; Zhang et al. 2024) (Table 1).

**TABLE 1:** Revised overview of disorder‐associated gut microbial patterns in sleep disorders.

Sleep‐related condition	Recurrent microbiota‐associated findings	Main biological correlates	Evidence type	Key limitation	Key supporting references
Insomnia	↓ *Bifidobacterium*, ↓ *Faecalibacterium*, variable reduction in *Lactobacillus*; relative increase in Proteobacteria and other stress‐associated taxa	Higher cortisol reactivity, possible barrier dysfunction, inflammatory tone	Primarily human observational studies	Strong confounding by stress, diet, psychiatric symptoms, and medication exposure	(Kumar 2022; Nie and Tian 2022)
Circadian rhythm disruption	Reduced microbial rhythmicity, altered temporal abundance patterns, enrichment of Enterobacteriaceae in some models/cohorts	Loss of host–microbe temporal alignment, inflammatory signaling, reduced SCFA rhythmicity	Human jet lag data plus preclinical circadian models	Time‐of‐sampling and rhythm‐related variation complicate interpretation	(Thaiss et al. 2014)
Obstructive sleep apnea	↑ *Prevotella*, ↑ *Streptococcus*, ↑ *Actinomyces*; relative depletion of barrier‐supportive taxa such as *Faecalibacterium*	Inflammatory activation, metabolic stress, neurocognitive associations	Human observational studies with supporting experimental models	Obesity and metabolic comorbidities remain major confounders	(Ko et al. 2019; Tang et al. 2024)

## 4 Experimental Models of Sleep Disruption and Causal Inference

Experimental models of sleep disruption provide an important framework for examining whether disturbed sleep can actively reshape the gut microbiome and whether those microbial changes can, in turn, influence neurobehavioral, inflammatory, and metabolic outcomes. This area is particularly valuable because it allows stronger inference about biological directionality than most cross‐sectional human studies, which often cannot determine whether dysbiosis precedes sleep pathology, follows it, or reflects shared upstream stressors (Sun et al. 2023; Wankhede et al. 2024). At the same time, most mechanistic evidence still derives from animal models, and direct translation to human sleep disorders remains incomplete.

### 4.1 Acute and Chronic Sleep Restriction

Animal studies consistently suggest that both acute and chronic sleep restriction can alter microbial diversity, reduce taxa associated with mucosal stability, and increase organisms linked to inflammatory or oxidative stress. In one mouse model, 72 h of SD reduced alpha diversity, lowered *Lactobacillus* and *Bifidobacterium*, and increased *Desulfovibrio*, changes that were accompanied by elevated circulating IL‐6, reduced tight junction integrity, and early endotoxemia (Yang et al. 2022). These findings support the view that sleep loss is not merely associated with dysbiosis, but may actively destabilize microbial ecology and gut barrier function under experimental conditions (Chen et al. 2023; Sun et al. 2023).

Human evidence points in the same general direction, although it is less mechanistically resolved. Short‐term sleep restriction has been associated with reduced abundance of SCFA‐producing bacteria, altered Firmicutes‐to‐Bacteroidetes balance, higher cortisol levels, and metabolic disturbances related to insulin sensitivity and appetite regulation (Hong et al. 2025; Zheng and Li 2024). However, the human literature remains limited in size and heterogeneous in design, and the magnitude, reproducibility, and time course of microbiome change after short‐term sleep loss are still not well defined. For this reason, experimental sleep restriction is better interpreted as biologically suggestive than as proof of a uniform dysbiosis pattern across all forms of human sleep loss (Rosén et al. 2022; Sun et al. 2023; Wu et al. 2026).

### 4.2 Bidirectional and Transfer‐Based Experimental Evidence

Experimental models also suggest that microbiome alterations may contribute to some downstream consequences of sleep disruption. Antibiotic‐induced depletion of the microbiota has been reported to worsen anxiety‐like behavior, cognitive impairment, and neuroinflammatory responses in sleep‐disrupted animals, suggesting that the integrity of the microbial ecosystem may influence host resilience to sleep loss (Wang et al. 2020; Yao et al. 2022). Conversely, experimental rescue paradigms, including metabolite replacement and fecal microbiota transfer, have been associated with attenuation of neuroinflammation and improvement in selected behavioral or physiological outcomes (Lin et al. 2024; Tung et al. 2024).

These findings are important because they move the field beyond descriptive association and begin to test functional involvement. At the same time, they should be interpreted with caution. Transfer studies and metabolite‐based rescue experiments support biological plausibility, but they do not fully reproduce the clinical heterogeneity of human insomnia, circadian disruption, or OSA. The strongest conclusion at present is that sleep loss can disturb the microbiome, and that experimentally altered microbial states can modify some consequences of sleep disruption, particularly inflammation, stress responsivity, and cognitive performance, without yet establishing a complete translational model for human disease (Pan et al. 2025; Withrow et al. 2021; Zhang et al. 2021).

### 4.3 IH Models and OSA‐Relevant Experimental Evidence

OSA is characterized by recurrent upper airway collapse, IH, sleep fragmentation, and substantial cardiometabolic stress. While clinical studies suggest that OSA is associated with gut dysbiosis, experimental IH models have provided more direct evidence that apnea‐like physiological stress can actively reshape the gut microbiome and promote downstream inflammatory and neurobehavioral consequences. This distinction is important because it shifts the discussion from descriptive association toward mechanistic testing under controlled conditions.

Rodent models of IH have shown increased intestinal permeability, reduced abundance of SCFA‐producing organisms, and expansion of taxa linked to inflammatory and oxidative stress, including *Desulfovibrio* and certain *Clostridium* species (Durgan et al. 2018). These changes are often accompanied by reduced butyrate availability, disruption of epithelial barrier integrity, and greater systemic exposure to microbial products. Rather than representing only a taxonomic shift, these findings suggest that IH may alter microbial metabolic output, mucosal defense, and host immune tone in parallel, which is more informative mechanistically than listing altered genera alone (Lv et al. 2023; Snodgrass and Velayudhan 2026; Song et al. 2024).

Evidence from microbiota transfer studies has further strengthened this interpretation. Fecal microbiota transplantation (FMT) from IH‐exposed mice into healthy recipients has been reported to induce hippocampal inflammation, anxiety‐like behavior, and impaired synaptic plasticity, indicating that microbiome disruption is not merely an epiphenomenon of OSA‐like stress but may contribute functionally to at least some of its downstream neurological effects (Badran et al. 2020). Experimental work has also linked IH‐related dysbiosis to LPS‐driven microglial activation and increased BBB permeability, supporting a model in which gut‐derived inflammatory signaling amplifies CNS injury (Ayyaswamy 2022).

The broader relevance of these findings lies in the interaction between OSA‐related dysbiosis, metabolic dysfunction, and inflammatory burden. Insulin resistance, hypertension, leptin dysregulation, and systemic cytokine elevation are common in OSA and may be intensified by gut‐derived inflammatory mediators and altered microbial metabolism (Tang et al. 2024; Tang et al. 2022). However, these relationships require cautious interpretation, because obesity, dietary pattern, and baseline metabolic disease can independently shape the microbiome and may confound both clinical and experimental observations. For that reason, IH models should be interpreted as biologically informative systems that clarify plausible gut–brain mechanisms, rather than as complete reproductions of human OSA pathology (Almendros et al. 2022; Lv et al. 2023; Patoine et al. 2026).

Taken at their strongest, these models support the microbiome as a mechanistically relevant component of OSA‐related inflammatory and neurobehavioral dysfunction. They do not yet establish microbiome‐based therapy as a clinically validated strategy in OSA, and direct human interventional evidence remains limited (Badran, Mashaqi, et al. 2020; Rodríguez‐Nogales and Gálvez 2020).

## 5 Chronobiology and Microbial Rhythmicity

The gut microbiome is not only defined by taxonomic composition but also by temporal organization. Microbial abundance, gene expression, and metabolic output fluctuate across the day in coordination with host feeding behavior, endocrine rhythms, and sleep–wake timing (Choi et al. 2021; Matenchuk et al. 2020). This chronobiological dimension is especially important in sleep research because it helps explain why circadian disturbance cannot be understood solely in terms of which taxa are present. The timing of microbial activity may be as important as microbial composition itself, particularly in relation to inflammatory signaling, metabolite production, and host clock regulation (Gutierrez Lopez et al. 2021; Zheng et al. 2025).

### 5.1 Diurnal Organization of the Gut Microbiome

Under physiologic conditions, the gut microbiome exhibits daily rhythmicity in both composition and function. Experimental work has shown that microbial gene expression follows circadian oscillations that align with feeding–fasting cycles and host rest–activity patterns, with metabolic and immune pathways varying across the 24‐h period (Thaiss et al. 2014; H. Wang et al. 2022). These oscillations are shaped by host signals such as melatonin, cortisol, nutrient availability, and autonomic tone, while microbial metabolites can in turn influence peripheral clock‐related pathways (Ramos Meyers et al. 2022; Yin et al. 2020).

This temporal framing is important because it moves the discussion beyond static dysbiosis. A person may not show dramatic taxonomic disruption at a single time point and yet still have substantial loss of microbial rhythmicity across the day. That is one reason why cross‐sectional microbiome studies may underestimate the biological relevance of circadian disturbance. Emphasizing this point helps distinguish the chronobiology section from the earlier disorder‐specific dysbiosis section and reduces repetition (Li et al. 2018; Ma et al. 2024).

### 5.2 Circadian Misalignment and Microbial Desynchrony

Circadian misalignment, whether driven by jet lag, shift work, irregular sleep schedules, or repeated light–dark disruption, can disturb microbial oscillations and weaken host–microbe temporal coupling. In a human jet lag model, circadian phase shift was associated with increased abundance of Enterobacteriaceae, altered microbial rhythmicity, and enrichment of pathways related to LPS synthesis (Thaiss et al. 2014). Animal studies using inverted light–dark cycles or related paradigms have reported reduced butyrate production, disrupted intestinal and central clock gene expression, and greater inflammatory and metabolic instability (Leone et al. 2015; Tofani et al. 2024; Wang et al. 2025).

More recent large‐scale human evidence also supports the relevance of this relationship. In a 2026 analysis of 6941 participants from the Lifelines Dutch Microbiome Project, lower alpha diversity was associated with poorer sleep quality, later chronotype, and greater social jet lag, while beta diversity was linked to both sleep quality and social jet lag. These findings do not establish causality, but they strengthen the argument that microbial variation is detectable across multiple human sleep and circadian dimensions, not only in small or highly selected cohorts (Wu et al. 2026).

The key point here is not merely that circadian disruption changes the microbiome, but that it may impair the temporal coordination between microbial signals and host physiology. This temporal desynchrony could influence sleep latency, neuroinflammatory tone, metabolic regulation, and stress responsivity even when total sleep duration is not the only variable affected. Presenting microbial desynchrony as a timing disorder rather than only a compositional disorder makes the section more conceptually distinct and more aligned with current chronobiology research (Bautista and López‐Cortés 2025; Zhao et al. 2026).

There is also an important interpretive caution. Much of the strongest evidence for microbial desynchrony comes from preclinical models, whereas human studies are fewer and often limited by sparse sampling across time. Even large cohort studies remain observational and cannot by themselves resolve temporal directionality or mechanistic mediation. This means that the clinical relevance of disrupted microbial rhythmicity is highly plausible but still incompletely defined (Heppner et al. 2024; Romanenko et al. 2025; Zhao et al. 2022).

### 5.3 Chrononutrition and Microbial Re‐Entraining Strategies

Because microbial rhythms are influenced strongly by feeding timing, chrononutritional approaches such as time‐restricted feeding (TRF) have attracted attention as potential tools for microbial re‐entrainment. In experimental models, restricting food intake to defined windows has been shown to restore microbial oscillations, improve SCFA rhythmicity, and reduce some of the metabolic consequences of circadian disruption (Daas and De Roos 2021; Fang et al. 2023). These findings suggest that microbial timing may be modifiable even when sleep timing or environmental conditions are suboptimal.

For the purposes of this review, it is important not to overstate these data as established sleep therapies. At present, TRF and related behavioral strategies are better viewed as translationally promising rhythm‐alignment tools than as proven microbiome‐based treatments for insomnia or circadian sleep disorders. Their value may lie in restoring synchronization between host behavioral cues and microbial metabolic timing, which could indirectly support sleep quality and metabolic stability (Machado et al. 2022; Xia et al. 2022).

This area is likely to become more important as clinical sleep medicine moves toward multidimensional intervention models that consider meal timing, light exposure, activity pattern, and microbial dynamics together rather than in isolation. This framing also helps the manuscript transition naturally into the section on translational barriers and future directions, where the need for longitudinal and time‐resolved human studies can be emphasized (Abou‐Khalil 2025; Wu et al. 2026).

## 6 Microbiome‐Targeted Interventions

**FIGURE 3:**
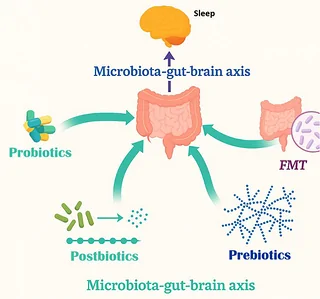
Microbiome‐targeted strategies for modulating the microbiota–gut–brain axis in sleep‐related disorders. Probiotics, prebiotics, postbiotics, and fecal microbiota transplantation may influence sleep‐related outcomes by reshaping gut microbial composition, microbial metabolite production, immune tone, barrier function, and gut–brain signaling. These approaches remain promising but should currently be interpreted as emerging adjunctive strategies rather than established stand‐alone sleep therapies.

Targeted modulation of the gut microbiome has emerged as a promising, although still early‐stage, strategy for improving sleep‐related outcomes. Because sleep disturbance can alter microbial composition and microbial activity can in turn influence inflammatory, endocrine, neural, and circadian pathways relevant to sleep, interventions aimed at restoring microbial balance have attracted growing interest (Lin et al. 2024; Wankhede et al. 2024). At present, however, the central question is not whether microbiome‐targeted interventions are biologically plausible, but whether their effects are sufficiently reproducible, clinically meaningful, and transferable across different sleep phenotypes and patient populations. The available literature remains uneven in design, endpoint selection, and mechanistic resolution (Cano and García Menéndez 2026; Lynch et al. 2025) (Figure 3).

### 6.1 Probiotics and Psychobiotics

Probiotics and psychobiotics have received the greatest attention in translational sleep research. Selected human randomized controlled trials (RCTs) suggest that strains such as *Lactobacillus gasseri* CP2305 may reduce stress‐related cortisol signaling and improve perceived sleep quality, while *Bifidobacterium longum* 1714 has shown favorable effects on sleep latency, resilience, and stress responsiveness in some populations (Nishida et al. 2019; Patterson et al. 2024). These findings are encouraging, but they should be interpreted as strain‐specific rather than class‐wide effects. They also require careful reading because improvements in perceived sleep or stress resilience do not always correspond to robust changes in objective sleep architecture (Chichlowski et al. 2022; Lin et al. 2024).

Preclinical studies provide additional mechanistic support. For example, *Lactobacillus reuteri* has been reported to reduce pro‐inflammatory cytokines, improve NREM sleep, and increase hypothalamic GABA‐related signaling in animal models (Y. Jiang et al. 2025). Even so, probiotic effects should not be generalized across species, formulations, doses, or clinical contexts. The current evidence supports selective biological promise, not a universal sleep benefit attributable to probiotics as a whole. This distinction is important because much of the literature still relies heavily on subjective sleep measures, small sample sizes, and relatively short intervention periods (Lin et al. 2024; Yu et al. 2024).

### 6.2 Prebiotics, Dietary Fiber, Synbiotics, and Postbiotics

Prebiotics such as inulin and galactooligosaccharides (GOS) can selectively support beneficial gut microbes and enhance the generation of metabolites relevant to host physiology. In animal studies, prebiotic‐enriched diets have been associated with improved intestinal barrier function, greater SCFA production, and increased hippocampal brain‐derived neurotrophic factor, changes that may support more stable and restorative sleep (Davani‐Davari et al. 2019). Human evidence is more limited, but early studies suggest that increased fermentable fiber intake may modestly influence fecal butyrate levels and selected sleep‐related outcomes (Hodgkinson et al. 2023). At present, these data are better viewed as mechanistically supportive than clinically definitive.

Synbiotic strategies, which combine probiotics with substrate support for microbial growth, may also be conceptually relevant in this setting because they attempt to improve both microbial viability and metabolic function. However, sleep‐focused evidence for synbiotics remains limited, and their role in sleep medicine is still largely exploratory (Zhang et al. 2025).

Postbiotic approaches, particularly those centered on butyrate, may offer a more controlled way of targeting microbial function without requiring administration of live organisms. Butyrate supplementation has been linked to reduced neuroinflammatory signaling and improved NREM sleep in preclinical models (Matt et al. 2018). This makes postbiotics conceptually attractive, especially where live microbial therapies may be less predictable or less stable. Still, most of the sleep‐specific evidence remains preclinical, and it would be premature to frame butyrate or related postbiotics as clinically validated sleep therapeutics (Haarhuis et al. 2022).

### 6.3 Fecal Microbiota Transfer and Microbiome Restoration Strategies

FMT has been explored as a means of restoring microbial diversity and function in models of sleep‐related physiological stress. In animal studies, microbiota transfer from healthy donors has improved cognitive and inflammatory outcomes in settings of chronic SD or IH, suggesting that restoration of a healthier microbial ecosystem can modify some consequences of disturbed sleep (X. Jiang et al. 2025). These observations are mechanistically informative because they support functional relevance of the microbiome rather than simple association alone.

Even so, FMT should be discussed with considerable caution in a sleep‐focused review. Its current relevance is experimental rather than routine, and the human evidence for primary sleep disorders remains extremely limited. It is therefore more accurate to present fecal transfer studies as proof‐of‐principle for microbial causality and reversibility than as near‐term therapeutic options for insomnia, circadian disturbance, or OSA (Chen et al. 2023; Lau et al. 2024).

### 6.4 Adjunctive and Disorder‐Specific Translational Opportunities

Microbiome‐targeted strategies are likely to be most useful as adjunctive rather than stand‐alone approaches. In insomnia, psychobiotic or prebiotic interventions may be most relevant in patients with prominent stress reactivity, inflammatory burden, or incomplete response to standard behavioral treatment. In OSA, microbial modulation could theoretically complement continuous positive airway pressure (CPAP) by addressing residual inflammatory and metabolic abnormalities that airway correction alone does not fully normalize (Lv et al. 2023; Wu et al. 2021). This framing is more realistic and clinically defensible than presenting microbiome therapy as a replacement for established sleep interventions.

Dietary strategies, including higher fiber intake, chrononutritional alignment, and broader dietary pattern optimization, may offer lower‐risk ways to influence microbial composition and metabolite production while fitting more naturally into long‐term sleep care. However, the translational literature remains limited by short follow‐up, small sample size, variable intervention design, inconsistent sleep phenotyping, and inadequate stratification of likely responders. What the field now needs is not another broad claim that microbiome interventions are effective, but better‐designed trials capable of identifying which formulations are biologically credible, which patients are most likely to benefit, and which sleep outcomes are genuinely modified (Chen et al. 2026; Sejbuk et al. 2024; Withrow et al. 2021). A consolidated comparison of the major microbiome‐targeted intervention classes, their proposed sleep‐relevant mechanisms, representative findings, evidence base, and key limitations is provided in Table 2.

**TABLE 2:** Mechanistic and evidence‐based overview of microbiome‐targeted intervention classes in sleep‐related research.

Intervention class	Proposed sleep‐relevant mechanisms	Representative findings	Evidence base	Key limitations	Key supporting references
Probiotics/psychobiotics	Modulation of stress signaling, inflammatory tone, gut–brain communication, and neuroactive pathways	*L. gasseri* CP2305 and *B. longum* 1714 show beneficial effects in selected human studies; preclinical studies support cytokine‐ and GABA‐related effects	Human RCTs plus preclinical data	Strain specificity, small trials, variable endpoints, and frequent reliance on subjective sleep outcomes	(Nishida et al. 2019; Patterson et al. 2024; Zhang et al. 2025)
Prebiotics and dietary fiber	Increased SCFA production, support of barrier integrity, and indirect immune and neuroendocrine modulation	Preclinical evidence is stronger than human evidence; some early human studies suggest modest benefit	Mostly preclinical, limited human data	Mechanistic plausibility currently exceeds clinical confirmation	(Bowers et al. 2022; Thompson et al. 2016; Thompson et al. 2020)
Synbiotics	Combined support of microbial supplementation and substrate availability	Conceptually relevant, but direct sleep‐focused evidence remains limited	Early exploratory rationale	Sparse sleep‐specific data and limited standardized evaluation	(Lau et al. 2024; Zhang et al. 2025)
Postbiotics, including butyrate	HDAC‐related signaling, neuroimmune modulation, barrier support, and reduced inflammatory signaling	Improved non‐rapid eye movement sleep and reduced neuroinflammation in experimental models	Primarily preclinical	Few sleep‐focused human intervention studies	(Fawad et al. 2022; Fukushima 2022; Szentirmai et al. 2019)
Fecal microbiota transplantation	Broad restoration of microbial ecology and microbial metabolic output	Functional improvement in experimental models of sleep deprivation and intermittent hypoxia	Experimental animal evidence	Very limited direct relevance to current clinical sleep practice	(Badran et al. 2020; Wang et al. 2021; Zhao et al. 2023)
Dietary and adjunctive microbiome strategies	Support of long‐term microbial stability, chronobiological alignment, and metabolic regulation	Potential role alongside standard care in insomnia and OSA	Emerging translational concept	Lack of standardized protocols, disorder‐specific trials, and responder stratification	(Bock et al. 2024; Xue et al. 2024)

## 7 Translational Barriers, Limitations, and Future Directions

The growing literature on gut microbiome–sleep crosstalk has established a biologically credible relationship between microbial ecology, inflammatory signaling, neuroendocrine regulation, circadian timing, and sleep physiology. At the same time, the field is still at a stage where mechanistic plausibility exceeds clinical readiness. Human studies have repeatedly shown associations between sleep disturbance and altered microbial composition, while preclinical models have provided stronger support for functional involvement through SD, IH, metabolite supplementation, and microbiota transfer paradigms. What remains less certain is whether these findings can be translated into stable biomarkers, reproducible interventions, or patient‐specific therapeutic strategies across real‐world sleep disorders (Pacheco et al. 2024).

A major challenge is that much of the current human evidence remains observational. Dysbiotic patterns have been reported in insomnia, circadian disruption, and OSA, but most clinical studies cannot determine whether microbial changes precede sleep pathology, arise as a consequence of it, or reflect shared upstream drivers such as stress, obesity, diet, medication exposure, or metabolic dysfunction (Hossain et al. 2022; Nie and Tian 2022; Pacheco et al. 2024). This uncertainty is especially important because several microbial signals described across sleep disorders are not disease‐specific and may instead reflect broader inflammatory or stress‐responsive states. Another limitation is the uneven quality and comparability of available data. Studies vary substantially in microbiome sampling strategy, sequencing platform, taxonomic resolution, metabolite measurement, and bioinformatic processing. Sleep assessment is similarly heterogeneous, ranging from subjective questionnaires to actigraphy and polysomnography, often without consistent alignment between microbial sampling time and sleep phenotyping (Park et al. 2026). This methodological heterogeneity makes cross‐study comparison difficult and weakens confidence in any single microbial signature as a clinically actionable marker. It also limits interpretation of intervention studies, where formulation, dose, treatment duration, baseline microbiota, and host dietary context differ widely across cohorts (Rodríguez‐Nogales and Gálvez 2020; Sivamaruthi et al. 2025).

Clinical translation is further complicated by strong interindividual variability. Baseline microbial composition differs across age groups, dietary patterns, metabolic states, medication exposures, and geographic settings, which means that a probiotic or dietary intervention that appears beneficial in one cohort may not produce the same effect in another. This is especially relevant for psychobiotics, where strain‐specific actions are often highlighted but not always tested under sufficiently comparable clinical conditions. A recurring problem in the literature is that positive findings in stress resilience or mood‐related endpoints are sometimes interpreted too quickly as direct evidence of sleep‐specific efficacy, even when objective sleep improvements are limited or inconsistently measured (Bautista et al. 2025; Van Hul and Cani 2026).

From a therapeutic standpoint, microbiome‐targeted strategies are probably best viewed as adjunctive rather than replacement approaches at this stage. Selected probiotics, prebiotics, dietary modulation, and chrononutritional interventions may have a role in patients with stress‐sensitive insomnia, circadian instability, metabolic dysfunction, or residual inflammatory burden, but they should not yet be presented as established stand‐alone treatments for primary sleep disorders (Li et al. 2018). In OSA, for example, CPAP remains the standard of care, while microbiome‐directed approaches are more realistically framed as possible adjuncts for inflammatory or metabolic comorbidity rather than direct substitutes for airway‐based therapy (Xue et al. 2024). Likewise, in chronic insomnia, microbiome‐oriented interventions may eventually complement cognitive behavioral therapy for insomnia (CBT‐I) in selected patients, but the supporting evidence is not yet strong enough to justify routine recommendation beyond exploratory or individualized contexts (Scott et al. 2025).

The next phase of the field will require better study design rather than broader claims. Longitudinal human studies are needed to determine temporal directionality, especially in people transitioning from normal sleep to chronic sleep disruption. Intervention trials need standardized sleep endpoints, deeper microbial and metabolomic profiling, and clearer disorder‐specific stratification. Time‐resolved sampling will also be important, particularly for studies of circadian disruption, because microbial rhythmicity may be missed entirely when only single time‐point sampling is used. Precision‐oriented frameworks integrating microbiome data with metabolomics, neuroendocrine markers, inflammatory profiling, sleep architecture, and host clinical phenotype are likely to be more informative than taxonomic analysis alone (Sivamaruthi et al. 2025; Yue et al. 2023).

Future work should also aim to identify which patients are most likely to benefit from microbiome‐based strategies. That includes determining whether therapeutic responsiveness differs according to inflammatory status, metabolic phenotype, stress reactivity, circadian misalignment, or baseline microbial composition. This kind of stratified approach is much more likely to produce clinically useful findings than continuing to search for one universal sleep‐promoting microbiome profile. A more mature translational framework will therefore depend not only on larger studies, but also on better phenotyping, better timing, and more careful matching between mechanism, intervention, and patient subgroup (Grosicki et al. 2020; Lin et al. 2024; Ma et al. 2024). To clarify the current clinical position of these approaches, Table 3 summarizes the translational readiness, most relevant sleep contexts, and major barriers associated with microbiome‐based strategies in sleep medicine.

**TABLE 3:** Translational readiness and major clinical barriers of microbiome‐based strategies in sleep medicine.

Strategy	Proposed clinical role	Current evidence status	Most relevant sleep contexts	Major translational barriers	Key supporting references
Probiotics/psychobiotics	Adjunctive modulation of stress signaling, inflammation, and gut–brain communication	Early human evidence with some positive findings, but inconsistent across strains and cohorts	Stress‐sensitive insomnia, mild sleep disturbance, selected OSA‐related inflammatory settings	Strain specificity, variable trial design, limited objective sleep end points, interindividual microbiome variability	(Liu et al. 2025; Zhang et al. 2025)
Prebiotics and dietary fiber	Support of beneficial taxa and microbial metabolite production	Mechanistically plausible, with limited and mixed human sleep data	Chronic insomnia, metabolic sleep disturbance, circadian instability	Weak standardization of interventions, limited long‐term trials, uncertain responder profiles	(Bowers et al. 2022; Sasaki et al. 2025)
Postbiotics/SCFA‐based approaches	More controlled targeting of microbial function without live organisms	Stronger preclinical support than clinical evidence	Neuroinflammatory sleep phenotypes, experimental sleep disruption models	Lack of sleep‐focused human trials, uncertain dose translation, incomplete mechanistic validation in patients	(Fawad et al. 2022; Fukushima 2022; Szentirmai et al. 2019; Wang et al. 2024)
Chrononutrition/**time‐restricted feeding**	Realignment of host–microbe temporal organization	Promising experimental and conceptual support, limited disorder‐specific clinical validation	Shift work, circadian misalignment, delayed sleep timing	Compliance, chronotype dependence, sparse time‐resolved human microbiome data	(Singh et al. 2025)
CPAP plus microbiome‐targeted adjuncts	Complementary reduction of inflammatory or metabolic burden in OSA	Conceptually attractive but clinically underdeveloped	Moderate‐to‐severe OSA with metabolic comorbidity	Few direct intervention trials, unclear additive benefit, confounding by obesity and diet	(Tang et al. 2024; Xue et al. 2024)
CBT‐I plus microbiome‐oriented support	Potential enhancement of treatment response in refractory or stress‐linked insomnia	Exploratory concept with limited direct evidence	Chronic insomnia with incomplete CBT‐I response	Insufficient combined‐trial evidence, difficulty separating mood benefit from direct sleep benefit	(Li et al. 2024; Qaseem et al. 2016; Riemann et al. 2023; Wang et al. 2025)

## 8 Conclusion

The gut microbiome has emerged as a biologically relevant component of sleep regulation, with growing evidence linking microbial activity to neuroimmune signaling, neuroendocrine balance, circadian organization, and host metabolic homeostasis. Across sleep‐related conditions such as insomnia, OSA, circadian misalignment, and experimental sleep loss, altered microbial composition and function have been associated with inflammatory activation, impaired barrier integrity, and disrupted production of metabolites involved in gut–brain communication. These observations support a bidirectional relationship between sleep and the microbiome, but they do not yet justify assuming that all reported microbial changes are causal, disorder‐specific, or clinically actionable.

Mechanistic studies have helped clarify how this interaction may operate. SCFAs, tryptophan‐related metabolites, immune mediators, vagal signaling, and HPA axis modulation all appear capable of shaping sleep‐related physiology under specific conditions. The strongest mechanistic support currently comes from preclinical models, particularly those involving SD, IH, microbial metabolite manipulation, and microbiota transfer. By contrast, most human evidence remains associative, and the biological direction, stability, and therapeutic relevance of many reported microbial signatures are still incompletely defined.

The translational potential of this field is promising but still developing. Psychobiotics, prebiotics, dietary modulation, chrononutritional strategies, and other microbiome‐oriented interventions may eventually contribute to more personalized care in selected sleep phenotypes, especially where inflammation, stress reactivity, circadian instability, or metabolic dysfunction are prominent. At present, however, these approaches are better viewed as emerging adjunctive strategies than as established stand‐alone therapies. Their clinical utility will depend on stronger evidence, clearer patient stratification, more consistent sleep phenotyping, and better characterization of microbial function beyond descriptive taxonomy.

Future progress will require longitudinal human studies, standardized and time‐resolved microbiome sampling, multi‐omic integration, and mechanistically informed intervention trials capable of distinguishing correlation from causal contribution. A more mature understanding of the gut–brain–sleep axis will likely depend on identifying which microbial pathways matter most, in which patient subgroups, and under which physiological or pathological contexts. The most meaningful advance for the field will not be the proposal of a single universal sleep‐promoting microbiome, but the development of biologically grounded and clinically testable models that connect microbial function to specific sleep phenotypes.

## Author Contributions

**Ghaleb Oriquat**: writing – original draft, writing – review and editing. **Shaker Al‐hasnaawei**: writing – original draft, writing – review and editing. **Hayjaa Mohaisen Mousa**: writing – original draft, writing – review and editing. **Malathi H**: writing – original draft, writing – review & editing. **SIYA Singla**: writing – original draft, writing – review and editing. **Samir Sahoo**: writing – original draft, writing – review and editing. **Vimal Arora**: writing – original draft, writing – review and editing. **Ashish Singh Chauhan**: writing – original draft, writing – review and editing. **Mehrdad Nourizadeh**: conceptualization, writing – original draft, writing – review and editing, supervision.

## Funding

The authors have nothing to report.

## Ethics Statement

The authors have nothing to report.

## Consent

The authors have nothing to report.

## Conflicts of Interest

The authors declare no conflicts of interest.

## Data Availability

Data sharing is not applicable for this article as no datasets were generated or analyzed during the current study.
